# Developing players for athlete leadership groups in professional football teams: Qualitative insights from head coaches and athlete leaders

**DOI:** 10.1371/journal.pone.0271093

**Published:** 2022-08-03

**Authors:** Gina Haddad, Donna O’Connor

**Affiliations:** 1 Sydney School of Education and Social Work, Sydney, Australia; 2 The University of Sydney, Sydney, Australia; Sheffield Hallam University, UNITED KINGDOM

## Abstract

Athlete leadership groups (ALGs) are a widely used yet under researched approach to leadership in professional sports teams. Athlete Leadership Groups (ALGs) represent a shared athlete leadership model whereby a small group of players are selected as athlete leaders and appointed to a formal ‘leadership group’ (i.e., an ALG) that shares team leadership responsibilities with the coach. Although athlete leadership has been linked to improved team outcomes, inadequately trained athlete leaders can have a detrimental effect on team functioning and performance. The aim of this study was to provide coach and athlete leader’s perceptions of the development opportunities that have been afforded to players to prepare them for their role in an ALG. Semi-structured interviews were conducted with 16 head coaches and 14 players from leadership groups drawn from 17 teams across four professional football leagues (i.e., Super Rugby, National Rugby League, A League and Australian Football League) in Australia and New Zealand. Results illustrate that athlete leaders benefit from developing enhanced understanding of leadership as a multidimensional relational process, recognising various leadership styles, preferences, and how to leverage their influence with teammates. However, it is evident this theoretical knowledge alone is insufficient to equip players for a professional team ALG role. Players need opportunities to practice their developing leadership skills in authentic and appropriately challenging situations with support and facilitation. Findings point to the importance of systematic, individually tailored leadership development that includes scaffolded, structured experiential learning and meaningful interactions with other successful high-performance leaders. Further, this study reinforces the value of guided reflective practice in leadership development and how this process can enhance learning and transfer from leadership development initiatives. Finally, this study adds weight to calls from other researchers for coaches to be consistently intentional in employing leadership development strategies.

## Introduction

Athlete Leadership Groups (ALGs) represent a shared athlete leadership model whereby a small group of players are selected as athlete leaders and appointed to a formal ‘leadership group’ (i.e., an ALG) that shares team leadership responsibilities with the coach [1–3]. In Australia and New Zealand ALGs play a central role in team management in professional football teams (in the context of this study professional football refers to Rugby League, Rugby Union, Soccer and Australian Rules Football) [1]. In addition to providing the type of on-field leadership (e.g., directing the play, motivating teammates, interacting with referees) commonly associated with a team captains’ role, these athlete leaders fulfil many of the responsibilities traditionally seen as the preserve of head coaches and assistant coaches [1, 4, 5]. ALGs serve as an important conduit between the coach and players, disseminate coaching messages and giving the players a ‘voice’. These leadership groups provide important input into training, workload, game strategy, team management, selection and recruitment decisions [1, 5]. ALGs are responsible for monitoring, mentoring, and supporting teammates, holding them accountable to team standards and enforcing discipline [1]. There is a prevailing expectation that ALGs will exert influence on teammates in a way that positively influences motivation and commitment to team goals [1, 6, 7]. In fact, many coaches regard ALGs as the *primary* architect of team culture, and it is this perception of an ALGs’ ability to influence teammates and create a high-performance environment that underpins their adoption of this leadership model [1].

This shift to a formal athlete leadership role (i.e., in an ALG), and all of the additional expectations and responsibilities this entails, requires players to develop additional skills if they are to fulfil the role effectively [3, 8–10]. Leading within a shared leadership structure (such as an ALG) requires highly developed relational and identity leadership skills [1, 6, 11, 12]. Leaders in shared leadership models (i.e., ALGs) need a sophisticated understanding of group influence processes, the ability to develop and embed shared mental models, and highly developed constructive feedback and conflict management skills [13–15]. In this context leaders must demonstrate flexible thinking, an awareness of their own biases, openness to different perspectives and the ability to engage in collaborative decision-making [16]. The developmental demands for shared leadership are such that some scholars argue the shift to shared leadership can exponentially increase the need for professional development of individuals who will become leaders in this new structure [11]. However, it is not clear if, or to what extent development practices in professional football have kept pace with the changing nature of some players’ roles as teams move to shared leadership approaches such as ALGs.

Inadequately trained, ill-equipped athlete leaders can have a negative impact on team functioning and performance [1, 17]. For example, Fransen and colleague’s experimental study with adolescent male soccer players demonstrated when athlete leaders thwarted teammates’ need for competence by behaviours such as responding to teammates’ mistakes with critical feedback or used body language or verbal expression that conveyed a loss of confidence in their team, teammates’ intrinsic motivation was undermined and performance deteriorated [18]. Research therefore underscores the importance of athletes being adequately prepared for leadership roles to optimise their potential to positively impact their team’s performance [9, 19–21]. From high school to elite sport contexts, studies continue to report that athlete leaders, such as team captains, receive little, if any, intentional leadership development, leaving them unprepared to fulfil the additional responsibilities required by their new role in the team [9, 21, 22]. In a recent study that exemplifies the evidence highlighting the lack of leadership development for athlete leaders, Voelker and colleagues found that although almost 90% of the US high school coaches in their study thought formal leadership development for team captains was ‘a good idea’, only 12.2% provided such education and training for their captains [22]. Furthermore, Cotterill and Cheetham’s 2017 qualitative study highlights that the absence of formal leadership development for players appointed to these important team leadership positions is also a concern at the elite level, with the professional rugby captains in this study reporting being forced to ‘take their development into their own hands’ [9]. It has been suggested that despite recognising the value of athlete leadership, coaches may not proactively provide leadership development for their athlete leaders because they lack the time, knowledge, and resources to do so [3, 10]. To this end, researchers have highlighted the need for evidence to inform athlete leadership development programs [3, 9, 10, 19]. Furthermore, researchers have noted the importance of context, pointing to the significantly different requirements athlete leaders face across sports, performance levels and cultures, suggesting there is a need for context-specific evidence to inform athlete leadership development initiatives [1, 3, 9]. However, much of the work in this area has focussed on the development of team captains [10, 23, 24] as opposed to the development of athletes to lead in *shared* athlete leadership structures such as ALGs, and been conducted with non-professional athletes [3, 6, 8, 23, 25, 26], or in non-football contexts [19, 27]. Consequently, there is little, if any, available research focussed on the development of athlete leaders for ALGs in the high-stakes professional football environment despite ALGs being widely used in this context. Effective leadership can make the difference between success and failure in elite sport and professional football coaches in Australia and New Zealand acknowledge that ALGs play a central role in the provision of this leadership [1]. Moreover, they recognise that an ALGs’ effectiveness hinges on the readiness of players for their expanded role as athlete leaders [1]. However, there is little context-specific literature available to guide the development of players for these crucial ALG roles. We have little insight into what support and leadership development opportunities ALG players are afforded in professional football teams. Exactly what leadership development looks like for players appointed to ALGs in professional football teams remains largely obscure. Little is known about coaches’ views on developing players for leadership roles within this specific shared athlete leadership structure at the professional level, the strategies they employ to do this or their perceptions about what works. Similarly, although previous research has highlighted professional football player’s feelings of being under-prepared for athlete leadership roles [9], player’s experiences and perceptions of their development for these ALG roles in professional teams remain unexamined. It is therefore both conceptually and practically important that we develop an initial understanding of the development of players appointed to these leadership groups in this context [1, 6, 9]. Given the evidence that capturing insight from sources with different perspectives and roles within the process contributes to more meaningful understanding [28], the current study sought coach and athlete leader’s perceptions of the development opportunities that have been afforded to professional football players to prepare them for their role in an ALG with the aim of providing initial insight into how players are developed for these ALG roles.

## Method

### Design and study context

Semi-structured interviews were considered the most appropriate methodology to explore this topic because of the lack of previous literature in the area and the need to gain initial understanding of the topic involved [29–31]. Interviews enabled participants to give their perspectives and interpretations of their experiences and provided an opportunity for participants to raise unanticipated topics and thereby provide insight in areas that had not previously been considered [29]. This approach is commonly employed in research that seeks to better understand athlete and coach experiences [3, 9] and is consistent with philosophical assumptions underpinning this research study [31, 32]. This study is grounded ontologically in relativism (i.e., that reality is created, mind-dependent and therefore multiple) and epistemologically in constructivism (i.e., that knowledge is constructed and subjective) [32]. Each coach and athlete leader has differing experiences of the athlete leadership development opportunities currently afforded to players in preparation for leadership roles within ALGs. It is therefore important to understand this topic from each individual participant’s lens [28]. Constructivism enables us to address the aims of this research project by capturing the meanings within each participants’ perception and considering how these align with other’s experiences of these athlete leadership development opportunities in their professional environment [32].

In selecting the sample for the current study, we therefore sought to achieve a balance of representation between coaches and the athlete leaders, while at same time capturing perceptions from the four different football leagues and a cross section of teams to achieve an understanding of leadership development for ALGs that was representative of the professional football landscape in Australia and New Zealand [28, 32]. Although this approach resulted in a sample which is larger than what may be commonly found in qualitative, interview-based studies, Braun and Clarke (2022) suggest that studies such as the current one with a broad aim, overall inductive and exploratory approach, and where analysis is focussed across the data set may require a comparatively larger participant group to achieve a richness of information to make a meaningful research contribution [33].

### Participants

Purposeful sampling was used to recruit sixteen male professional football head coaches (age *M* = 48.8 years, 37–62 years) and fourteen male professional football players (age *M* = 29.5 years, 25–36 years). This sample was recruited from 17 teams across the 4 professional football leagues in Australia and New Zealand (Inclusion criteria for coaches required; a) they were currently or had been recently employed full-time as a Head Coach in National first tier football league in Australia or New Zealand and b) had experience using an ALG (National Rugby League [NRL] n = 5, Australian Rules [AFL] n = 4, Super Rugby n = 4, and A League [football] n = 3). Inclusion criteria for players required they were currently a member of an ALG in a professional football team (NRL n = 2, AFL n = 6, Super Rugby n = 4, and A League n = 2). Experience at the professional level ranged from 2–15 years (*M* = 5 years) for coaches and 6–18 years (*M* = 10.2 years) for players. The participants were recruited either by a direct approach (via email) or via liaison with the authors’ industry contacts. Ethical approval to conduct the study was provided by the University of Sydney Ethics Advisory Committee and all participants provided informed written consent prior to participation.

### Data collection

Each coach and athlete leader were interviewed by the first author. A total of 30 semi-structured interviews (16 coaches and 14 athlete leaders) were conducted. Wherever possible interviews were conducted face-to-face (n = 20). When geographical constraints precluded this, phone (n = 9) or Skype (n = 1) interviews were used. An emphasis was placed on developing a rapport with participants during the various communications that preceded their interview (i.e., conversations undertaken to establish their willingness to participate and scheduling a mutually convenient interview time). The level of engagement, natural flow of conversation and personal disclosure indicated that these measures were effective. Care was taken to ensure that phone interviews were scheduled to ensure the participant had ample time and would be in a relaxed and private setting. The interviewer used verbal conversational cues to encourage the flow of the conversation and the increased senses of anonymity afforded by not being face to face appeared to contribute to more intimate disclosure, such that several of the phone interviews were the longest and most in-depth [34, 35]. The average duration of the interviews was 57 minutes. Interviews were audio recorded on a MP3 storage device with participant permission.

Two versions of a semi-structured interview guide were used to elicit information from the participant coaches and athlete leaders. These were developed by the first author, drawing on informal discussions with coaches and athlete leaders and informed by relevant literature. The interview guides were reviewed by an experienced academic and piloted on a head coach that was also a recently retired athlete leader, who was not part of the current study. Interview questions were designed to elicit participants’ perspectives of the current practices employed to develop players for ALG roles in professional football teams (e.g., Coach- Can you describe the leadership development that players receive for their ALG role? How well do you think this prepares players for those leadership roles and why? Which leadership development activities work best and why do you think that is? Athlete leaders- What training or development did you receive in preparation for your role in the ALG? In your experience, what leadership development was the most helpful and why?). Follow-up questions and probes were used to clarify a participant’s response or achieve more depth when necessary (e.g., Can you explain how that particular development activity helped you in your ALG role?) Questions were presented in a conversational manner designed to facilitate the natural flow of the interview. While each interview broadly followed the interview guide, it was employed with flexibility to enable the interviewer to pursue responses outside of the specific questions.

### Data analysis

Interviews were transcribed verbatim then imported to NVivo 12 Computer Assisted Qualitative Data Analysis Software (Qualitative Research Solutions International) for data management and analysis. The recognised 6 phases of reflexive thematic analysis (indicated below in italics) advocated by Braun and colleagues [33, 36–38] were used to analyse the data from the interviews. In the first *‘familiarisation’* phase, the first author immersed herself in the data, repeatedly reading and re-reading the interview transcripts through the lens of the research question to become intimately familiar with the data [38]. At this stage initial analytical thoughts and responses to the data were recorded in a separate notebook rather than the transcript margins so as not to constrain future coding. The second phase involved *generating codes*. A ‘label’ or code was allocated to all sections of the data that were identified as being meaningful and relevant to the research question. Transcripts were first coded manually using the comment function in Word and then as nodes in NVivo [38]. This process was approached from a primarily inductive, ‘bottom-up’ perspective, meaning that the analysis was data-driven and exploratory, in line with the purpose of this study [33, 36]. During this phase the first author engaged interpretatively with the data, seeking to explore both explicit (i.e., semantic) and implicit (i.e., latent) meanings, ideas, and assumptions in a systematic approach. Coding was open-ended, organic and inclusive [36–38]. Data extracts were coded at both a semantic (i.e., close to participant’s words and meaning) and latent (i.e., more interpretive) level as was considered appropriate and relevant to the research question [37, 38]. For example, ‘observing other HP leaders’ represents a semantic code, whereas the code ‘understanding group-based identity influence processes’ was a more interpretive (i.e., latent) code, reflecting the intersection of participants accounts, the research question and the researcher’s theoretical lens [36–38].

Similarly coded extracts were then gathered together to *construct candidate themes* in phase three. These were preliminary, ‘prototype’ themes that aimed to capture a pattern of meaning across the data set [33, 36]. For example, the codes ‘observing other HP leaders’, ‘mentoring’, ‘relatable HP leadership stories’, and ‘deliberate HP leadership social learning opportunities’ were ‘clustered’ together to form the candidate theme ‘Learning from Others’. Several iterations of a thematic map were used to assist in visualising the relationships between various codes and potential themes [36]. In consultation with the second author, phases four and five of the data analysis involved first *reviewing candidate themes* in relation to the coded extracts and the entire data set to assess validity and then *naming and defining* each theme [38]. During the review process it was identified that the prevalence and importance in relation to the research question of the sub-theme ‘Guide Reflective Practice’, which had initially been conceptualised as fitting within the broader theme of experiential learning, warranted promotion to a stand-alone theme [38]. Consistent with Braun and Clarke’s reflexive TA approach, codes and themes were determined by meaningfulness and importance in relation to the research question as opposed to frequency [33]. Once we had settled on theme names that captured the central organising concept of each theme, a brief paragraph was developed that provided a clear definition of each theme and delineated the themes [36–38]. The sixth and final phase was constructing a narrative *report* outlining the specifics of each theme in relation to the research question and relevant literature. In this phase the data extracts that best reflected the themes and would help to tell the story of the data were selected. It is important to note steps 1–5 were undertaken in as an inductive and iterative process, which involved deepening engagement with the data and constant revision and refinement of themes [33, 37, 38].

### Trustworthiness and reflexivity

The measures employed to improve the quality of this investigation are based on the relativist approach to conceptualizing validity in qualitative research advocated by Smith and Sparkes [31]. The sample included two perspectives (i.e., coaches and athlete leaders) and participants with experience of ALGs at the professional level from different football leagues, and clubs were purposively recruited to attain substantive contribution and width in relation to the research topic [28, 31]. In conducting the interviews, the first author shared her common experience of high-performance sport and spent time developing a rapport with participants. In conjunction with confidentiality measures which assured participants of anonymity, these measures established a high level of trust between the interviewer and participants which contributed to the depth of participant responses [29]. The second author acted as a critical friend [31] throughout the data analysis process, not with the aim of reaching coding consensus but rather to challenge the first author’s assumptions and interpretations in relation to the data and thereby encourage reflexivity. The first author also maintained a reflexive journal for the duration of the research project [31–33]. Included in the diary were details of the various methodological decisions, rationales, and personal reflections. Together these reflexive measures resulted in the ongoing reconfiguration of codes and themes over the course of the project which enhanced the coherence of the resulting analysis [28, 32, 38]. The inclusion of verbatim quotes in the discussion of the results facilitates the individual interpretation and application of results to the reader’s personal context [31, 38].

## Results and discussion

The aim of this study was to provide coach and athlete leader’s perceptions of the development opportunities that have been afforded to players to prepare them for their role in an ALG. Inductive analysis resulted in the development of four higher-order themes: Understanding leadership; Experiential learning; Learning from others; and Guided reflective practice. Each of these themes, which consist of multiple analytic observations from the data that are connected by a ‘central core meaning’ [33, 37, 38], are discussed below in relation to existing literature.

### Understanding leadership

Coaches and athlete leaders highlighted how being appointed to an ALG required players to reach a new, more nuanced understanding of leadership and to reconceptualise their role and identity within the context of the team. Participants acknowledged that players appointed to ALGs typically lacked fundamental knowledge about leadership and influence: *“They’re not professional leaders*, *just good at football*, *they don’t understand what it means to be a leader*, *or how they can have influence”* (C12). In becoming an athlete leader, they needed to rethink their primary responsibility beyond *“just being there to play footy”* and develop an understanding of “*the impact a leadership group can have”* (P1) on teammates. Most coaches recognised that players appointed to ALGs needed *“education around what a leader is and what leadership looks like in their organisation”* (C3). However, the difficulty in balancing development needs and performance in elite sports settings has been previously identified [1, 19, 39] and several coaches acknowledged that developing their ALGs’ understanding of leadership had not been prioritised: *“I haven’t put enough effort into exposing the leadership group to different philosophies and ideas of leadership*, *structured*, *formalized development which would allow them to better understand and establish their leadership style”*(C13). Although this was something of an exception, a small number of athlete leaders reported receiving no formal preparation for their role in an ALG: “*There was definitely no training*, *nothing to prepare me for that [leadership role]”* (P12).

Most teams provided, at a minimum, some type of *“learning to lead program”* (C4), an introduction to leadership, customarily delivered by an external consultant in a traditional classroom-type approach (i.e., lectures, presentations, small group activities) which aimed to help athlete leaders develop core leadership skills and “*understand what leadership is*, *and different leadership styles*” (C5). The scope and duration of these programs varied between teams; however, the content typically included a strong focus on athlete leaders *“understanding their own leadership style*, *their behaviours*, *how they can lead*, *what their strengths are”* (C12). This type of ‘knowledge of self’ has long been recognised as the basis of all leadership development [40] as self-awareness is central to a leaders’ ability to leverage their influence and shape the behaviour and attitudes of followers [41]. Participants in the current study indicated personality profiling [19] and 360 feedback processes assisted them in developing the self-understanding [42] necessary for their growth as an athlete leader: *“The coaches gave all the players a survey to rate our strengths and weaknesses*, *it was good to see what everyone else sees as my leadership style and my contribution and to see where I can improve as a leader”* (P5). These feedback processes were perceived to impact leadership development most when athlete leaders were provided with one-on-one coaching (from an experienced leadership facilitator) to facilitate the interpretation and application of this new knowledge of ‘self” and how others perceived them [43]. Participants believed it was beneficial for the entire team to complete personality assessments, so athlete leaders had a clearer understanding of each player’s personality traits and leadership preferences [19, 43].

Our findings reinforce previous work which has suggested a leader’s ability to understand their leadership capability is a core component of effective leadership [44]. As the excerpt below illustrates, developing self-awareness was central to athlete leaders understanding how aspects of their personality/behaviour influenced their leadership impact; “*My biggest thing that the leadership guy identified was the balance between being liked and being respected*. *That was something I really had to work on*. *That was the hardest and most challenging thing for me*.*”* (P11). It was clear that when athlete leaders understood their leadership style and strengths, they gained confidence in their leadership ability and were more able to leverage these strengths in a way that optimised their influence on teammates:

*By them teaching us more about ourselves, about who we are, what type of person we are, what type of leader, we’ve got more self-awareness in terms of being able to know our effect or impact on players around us…how we can get the best impact to get a desired outcome with a particular player…it’s just empowering*.*(P3)*

Importantly, when athlete leaders participated in training that developed their understanding of leadership as a multidimensional interaction characterised by individual leader and follower preferences, they were able to recognise diverse leadership needs and preferences of teammates [45]. This enabled them to modify their leadership behaviours accordingly and effectively ‘personalise’ their leadership approach [46]: *“It goes back to understanding individual personalities and leadership preferences… especially now we got a lot of Polynesians boys*. *They don’t take too well to that kind of leadership*. *Yeah*. *You go at the messages differently to individuals”* (P9). Athlete leaders in the current study consistently identified this nuanced understanding of individual leadership styles and preferences (including cultural sensitivities) as one of the most important learnings from their leadership development activities, reinforcing the findings of Cotterill’s leadership development intervention with elite cricketers in which participants highlighted developing their understanding of individual differences and how to modify their leadership approach was a valuable outcome of the intervention [19]. As athlete leaders grasped these concepts, they came to understand there was no singular ‘right’ way to lead and their confidence to lead authentically grew: “*I think authenticity is my biggest one*, *people just knowing who they are and understanding they don’t need to try and change to be a leader”* (P3).

Athlete leaders in the current study reported that the content delivered in these *‘learning to lead’* type programs not only challenged their self-concept (as they came to understand how they were perceived by others) it often challenged their existing conceptions of leadership more broadly. More specifically, athlete leaders acknowledged they often came to the program with a very narrow and ‘traditional’ (i.e., tough, masculine, authoritarian, vocal) view of the type of leadership required in a professional football team. Leadership development helped them to understand that relationships underpinned their ability to influence teammates, which led to an increased focus on caring for and building connections with teammates, as this athlete leader explained:

*Even two years ago I would have never thought having those personal relationships…making those personal connections was so important…when I came into footy it was pretty much just speak when you’re spoken to*, *you’d cop a spray*, *it was pretty autocratic*, *not much in the way of support*, *you’re here for footy just get on with it*, *that’s the thing we [the ALG] got wrong before but xxxx [the leadership facilitator] has been very helpful there*, *in how we are towards the playing group*, *now I understand you need to spend the time*, *build those relationships*, *show you care*, *… you can see a difference in the attitude of the person towards you when you give them feedback*, *the reception you get*, *when you have built that stuff behind the scenes*, *before I couldn’t see why I just couldn’t tell a player they weren’t doing their role and have no hard feelings*, *now I realise to be able to talk to each other like that you need to have something deeper behind it*, *because then you get each other in a subconscious way*. (P1)

Implicitly, participants appeared to reach an understanding of the role of empathy in leadership and recognised their influence was proportionally greater when they had invested in developing strong relationships with the teammates they sought to lead. Although previous research has identified relationship building, interpersonal and communication skills as core skills required by captains in professional rugby teams [9] participants in the current study placed particular emphasis on understanding the importance of strong relationships as a foundation for leadership influence. In this high stakes, high-pressure environment these professional players perceived close, caring personal relationships to be the foundation of their ability to effectively influence their teammates.

### Experiential learning

Coaches and athlete leaders in the current study identified core leadership skills that players appointed to ALGs needed to develop in order to fulfil their role. These included: providing peer feedback, having difficult conversations, managing conflict, public speaking, presenting, running meetings, contributing to management meetings etc. There was agreement among participants that these skills were most effectively developed through facilitated experiential learning. Coaches and athlete leaders agreed that much of the development of athlete leaders relied on “*learning on the job”*(C6) and “*learning from experience”* (P1). This experiential learning was a mix of unmediated and facilitated experiences. At times, elements of an athlete leaders’ development occurred organically, as a result of exposure to situations that they would not normally encounter as a player (i.e., when not a member of an ALG). For example, it was clear that including athlete leaders in discussions and meetings at a managerial level meant they developed more of a macro-view of the organisation (as opposed to a more self-focussed player view). They came to understand the broader impact and ramifications of various behaviours (e.g., off-field incidents leading to reputational damage, loss of sponsorship, memberships) which clarified their responsibilities and helped them to understand how they could add value as a leader:

*I was a bit naïve*, *as a footballer outside the leadership group you don’t get a full understanding of what’s actually required …when you’re in the leadership group you’re exposed to a lot more conversations*, *meetings and you become a lot more aware of how clubs run*, *how important memberships*, *sponsorship*, *and donors and all that stuff are*. *It really puts it into perspective… as a leader you’ve got an understanding of how important it is to drive the group… you actually take your job a little bit more seriously… you understand the flow on [effect] of poor results and stuff-ups*. (P4)

A small number of coaches took the view that athlete leaders “*just got better by doing it*” (C16). These coaches believed that unmediated ‘experience’ was all the leadership development players required. This is at odds with the prevailing view of researchers who argue that athletes will not become effective leaders simply by being appointed to a leadership role, without intentional leadership development [10, 19, 47]. Research underscores the importance of providing developing leaders with opportunities to experience leadership in meaningful, authentic situations and results from the current study reinforce this view. It was apparent that athlete leaders in professional football teams need to experience leadership in authentic situations and be supported to translate the learning from each experience [19, 44, 46, 48]. Although there was agreement among participants that a degree of ‘consequential’ learning [49] occurred simply by being in a leadership role, coaches with extensive experience of ALGs emphasised the importance of deliberating opportunities for leadership development, as opposed to relying on athlete leaders learning organically:

*Most of it it’s about experience and learning on the job*, *but there’s another bit that’s about being supported within a structured program…we find that really valuable… putting in place the development part for each individual*, *most days I say*, *"Where’s the opportunities*?*"… Should I have given an opportunity to someone there*, *instead of me speaking*?*" We put them in as many of those positions as we possibly can… It might be a 30-minute training session that they conduct*, *or a briefing on the bus before we go to a game … those moments allow your leadership group to grow and gain confidence*. (C9)

The volume and frequency of these authentic leadership ‘practice’ opportunities was considered important, however pedagogical research has highlighted the significance of ‘design’ in experiential learning [49] and coaches in the current study reinforced that experiential learning was most impactful when it was “*carefully planned”* (C1) and “*individualized”* (C5). Athlete leaders found real-life, learner-centred assignments, developed collaboratively with the team’s leadership facilitator/coach particularly useful in targeting identified gaps in their leadership skills and building confidence:

*He [facilitator]*, *checks in with each of us*…*he’ll say what’s your goal this month that you want to focus on about leadership*, *what are you working on getting better at*, *that sort of thing*, *we give him a goal every month of what we want to get out of it and we chat about it with him and he’ll give us like a task targeting that*, *my one for this month is getting more comfortable presenting in front of the group*, *it really helps you get more confident at those things*. (P11)

This type of action learning undertaken as ‘on-the-job’ ‘stretch’ assignments can accelerate leadership development and provide the most enduring learning [50]. While coaches recognised that athlete leaders needed to be ‘stretched’ in order to ‘grow’ as leaders, they were cognisant that leadership development assignments should be progressive and carefully aligned with the athlete leaders’ readiness. Most coaches interviewed recognised the importance of scaffolding the more challenging leadership tasks, to ensure athlete leaders had the skills and confidence to execute them [3, 19]:

*If it’s a potentially tricky conversation they [athlete leader] need to have*, *I maybe have to help them with some strategies*, *to guide them and say*, *"Now*, *you go home and have a think about it and tell me how*… *If you had to do it in an hour*, *what you would do*.*" Then we can piece it together*. *It’s guiding*, *mentoring but helping them grow*. (C7)

In this way, coaches in the current study appeared to implicitly understand the importance of fostering efficacy beliefs in developing athlete leaders at the professional level, something that has been previously identified in a leadership development intervention with elite cricket players transitioning to captaincy roles [21]. Some teams incorporated video-assisted feedback to enhance player’s learning from experiential leadership assignments. For example, a developing leader may be filmed conducting a team meeting or delivering feedback to a teammate and this footage is used in the debriefing afterwards: *“We’ll always video that*, *then they’ll [athlete leader] sit down with the leadership facilitator and get feedback on the way they did it*, *they improve from that*, *that’s where we find the value of the video”* (C6). The utility of video augmented feedback in leadership development has been recognised in other contexts (e.g., nursing) and coaches believed this assisted athlete leaders to realise things about their leadership approach that they would not otherwise identify through feedback alone [51].

One strategy that teams employed to provide developing athlete leaders with authentic, but developmentally appropriate experiential leadership learning opportunities was to implement emerging leaders’ programs. Identified potential future athlete leaders participated in structured leadership programs that operated a tier below the ‘senior’ ALG:

*We always run an emerging leaders’ group*, *it’s like the next crop coming through*. *I was in that for two years before I was in the leadership group*, *probably once a month or once every two months I would actually get to go into the leadership meeting*, *just to see how it all runs in there… it was good to be exposed to that sort of stuff early… when you get into the leadership group*, *you’re already prepared*. (P4)

Developing ‘bench strength’ to ensure a pool of ‘ready now’ leaders is a recognised strategy in corporate and military sectors to inoculate organisations against the loss of leadership talent and provide continuity [52]. Our results indicate that professional football teams employ tiered emerging leader’s programs in this way to facilitate succession. In high risk/high stakes environments there is often a trade-off between providing authentic development opportunities for future leaders and not compromising the performance outcomes of the organisation [52, 53]. In a professional sport setting emerging leaders’ programs were perceived to help teams to balance this ‘risk’.

Coaches and athlete leaders believed emerging leaders’ programs were most effective when they were closely linked to the senior ALG. Using practices that mirrored the accepted HR professional development practices of ‘job-rotation’ and ‘shadowing’, some teams immersed emerging leaders in the senior ALG on a rotational basis to expose them to the level of decision-making and leadership responsibilities required [52, 54]. There was consensus these measures helped ensure emerging athlete leaders were well-prepared to make a seamless transition to the senior ALG when required.

### Learning from others

Athlete leaders took important learnings away from observing the actions and interactions of other leaders (i.e., athlete leaders and coaches): “*For me it’s all about learning from and observing the best… "Standing on the shoulders of giants" is a great quote…just watching how the leaders went about it… learning from them…stealing their best principles”*(P10). Observing the reaction and consequences of another leaders’ communication style and interpersonal approach prompted athlete leaders to reflect on the effectiveness of various approaches:

*We had an example where our coach came down hard on this guy at half time*. *He’s a Polynesian*, *so that approach doesn’t work*. *Afterwards the player was like*, *" I didn’t like how the coach actually made me feel worse and play worse*, *but if he delivered it a different way*, *it would have helped me*.*" Maybe if he’d [coach] done it individually*, *pulled him aside*, *and lowered his voice a bit instead of standing over him and spraying him in front of the whole group*. (P8)

At times coaches and leadership facilitators engineered more deliberate, structured opportunities for developing athlete leaders to observe and/or interact with other leaders (e.g., guest speakers, visiting other leaders and observing them in their environment, mentoring). The general consensus from the athlete leaders was that they learnt more “*from real leadership stories rather than just theory”* (P3). In particular, athlete leaders connected strongly with “*real-life leadership examples”* (C7) from high performance environments (i.e., sport, military). Credibility was critical, athlete leaders were unlikely to engage with speakers/other leaders/mentors that failed to demonstrate “*genuine understanding of high performance”* (C5). Importantly, formal learning environments were less likely to foster engagement; “*As football players if there’s one thing we don’t like its meetings and power point presentations… you’d sit in a room and your mind wanders and you think about other things”* (P11). Informal and interactive approaches (with guest speakers and in-situ ‘visits’ to learn from other leaders) were seen to be more engaging and less intimidating, and therefore more likely to encourage questions and discussion that enhanced learning.

Reflecting an awareness of the recognised potential of mentoring to promote leader development, participants in the current study reported that most athlete leadership development efforts included some form of mentoring [26]. How and to what extent mentoring was employed by teams in their endeavour to support and develop their athlete leaders varied. Mentors were sometimes formally appointed, while some athlete leaders were left to source their own mentor. The consensus was that well-matched, motivated mentors could add considerable value to the development of an athlete leader, serving as a sounding board, critical friend and source of practical advice: “*He’s an ongoing mentor… I’ll talk to him regularly… "How should we handle this; how would you handle this*?*"* (P10). However, athlete leaders cautioned that mentoring was sometimes included in development programs as an afterthought, with insufficient consideration of the nuanced planning it necessitated, and the importance of athlete/mentor fit. Importantly, our results highlight that mentors in this environment could be a distraction if they lacked understanding of the high-performance environment, the athlete leader did not relate to them on a personal level, or they had the wrong motivation: *“It’s a good idea but you just have to make sure that the mentor isn’t just there to get the inside gossip… my guy was just a fan”* (P2). Participants indicated mentoring structures within emerging leaders’ programs benefited (i.e., efficacy beliefs, leadership skills) both mentors (i.e., senior ALG players) and mentees (emerging leaders) and worked best when players were supported to formalise their own mentor/mentee relationships.

### Guided reflective practice

A key finding from our study was the central role that reflective practice was perceived to play in the development of athlete leaders in these professional teams. More specifically, there was consensus among participants that the learning and transfer from leadership development experiences (i.e., on the job leadership assignments, guest speakers, visits to observe and interact with other leaders) was significantly enhanced when supported with guided reflective processes [19, 53, 55, 56]. Research in other fields indicates that a developing leader left to their own devices may arrive at an inaccurate decision, construct inaccurate knowledge, or develop ineffective or unsafe leadership practices [44]. Participants in the current study appeared to share this view and there was an awareness that developing athlete leaders needed support to make sense of the leadership experiences and education they were exposed to [19]. Consequently, there was a strong focus on guided reflection within leadership development initiatives, typically undertaken with a leadership facilitator. Guided reflection was used to encourage athlete leaders to scrutinise their leadership practices and identify areas for improvement: “*We debrief the task; asking* ***them*** *what was good*, *what was bad…how did you handle that*, *how did you think it went*?*”* (C8). Athlete leaders recognised this facilitation was key to achieving reflection that extended beyond the superficial and addressing the uncomfortable truths about what was impeding their growth as leaders [57]. They expressed an awareness of the benefit of the leadership facilitator *‘challenging them a lot’* (P8), asking the difficult questions, *‘hitting them straight between the eyes’*, believing that this *very honest*, *upfront* approach enabled the facilitator to encourage a deep, honest self-reflection. Although participants acknowledged that guided reflection could be a potentially uncomfortable experience, there appeared to be widespread recognition in the value of this process. Robust performance-based feedback is commonplace in these professional teams. As players, these athlete leaders were accustomed to receiving and observing honest and at times confronting feedback on their technical, tactical and physical performance. Implicitly, they appeared to not only be receptive to this type of feedback but to actually embrace it because they saw it as central to realising their potential. Because of this, when they became athlete leaders, they tended to approach their leadership development with the same perspective. As developing athlete leaders, they were open to being challenged if it would help them to improve. There was a prevailing view that a skilled facilitator could ask the difficult questions necessary for players to reflect on their development and performance thoughtfully and honestly as leaders, to explore alternative perspectives and to prevent unhelpful avoidance behaviours that would constrain their development as leaders.

Learning from interactions with other leaders (i.e. guest speakers and in-situ visits to observe other high performance leaders at work) was similarly optimised when athlete leaders had the opportunity to work with knowledgeable others (i.e., coach, leadership facilitator) afterwards to reflect, and explore different leadership approaches and consider how they might be applied in their context [48, 57]:*“We always found it helpful after those things talking with the leadership guy about them and seeing what ways that we could incorporate whatever we learned moving forward*” (P12). Guided reflection helped developing athlete leaders resolve tension between what they thought, what they observed from other leaders and the information from formal leadership training. This support ensured they did not simply reject information that was not aligned with their existing beliefs [57]. It enabled them to engage with conflicting ideas which is necessary to achieve the refined understanding of leadership necessary for this complex shared leadership approach [11].

## General discussion

The findings from this study make two important contributions to current understandings of athlete leadership development and sport leadership more broadly. First, our results provide initial insight into the current leadership development practices used to prepare players in professional teams for leadership roles in this novel and increasingly popular ALG approach to team leadership. Secondly, the findings from this study extend our understanding of coaches’ and athlete leaders’ perceptions of the utility and impact of various pedagogical approaches (e.g., traditional classroom style, experiential learning, social learning, and guided reflective practice) to athlete leadership development in this high-stakes, high-pressure environment.

Although ALGs are widely used in professional football teams in Australia and New Zealand, until now we have had little, if any, insight into what training or development players are afforded to prepare them for their role in an ALG. It is clear from our results that not all players appointed to ALGs are provided with the same level of leadership development. Our findings illustrate that approaches to athlete leadership development vary considerably between different teams. While previous research has identified that the development of athlete leaders is often neglected and ‘left to chance’ [3, 9, 10, 21], this was uncommon in the current study. Although a small number of players reported being initially appointed to an ALG with very little formal leadership development, most indicated that they had been afforded some degree of formal leadership development support. However, it was apparent there is considerable diversity in how teams approach the development of players for ALGs. For quite a few teams it was common for leadership development to only be programmed into the training week during the pre-season period. At the other end of the spectrum, it was clear from our results that some teams prioritise the development of their athlete leaders to the extent that it is placed at the very centre of their football program. Reflecting researchers calls for deliberate, well-designed athlete leadership development programs, these teams employed leadership facilitators who were embedded in the football program and delivered comprehensive, structured leadership development for their ALG [3, 9, 10, 19]. Importantly, in these teams, leadership development was timetabled into the training schedule every week throughout the entire competitive season as opposed to being limited to the pre-season. Many of these teams also provided tiered emerging leader’s programs whereby high potential future ALG leaders were identified and then engaged in a structured leadership development program designed to expose them progressively to developmentally appropriate and increasingly challenging real-life ALG situations and responsibilities. This type of structured support for emerging leaders as they navigate the ‘step-up’ to a senior leadership role has been previously identified as being critical to effective leadership development [55].

Overall, there was consensus among participants that teams using ALGs should prioritise leadership development for players appointed to the ALG and those likely to become ALG members in the future. It is noteworthy that on reflection, even coaches in the current study that had not previously prioritised athlete leadership development to this extent, recognised this as a shortcoming and expressed an intention to remedy this in the future. These findings underscore the need for intentional athlete leadership development. It is evident that ad hoc, piecemeal type offerings are insufficient to prepare players for ALG roles in professional teams, and the best results are realised when leadership development is structured and systematic.

Our results illustrate that leadership development for ALGs in many professional football teams extends beyond just the traditional classroom-based leadership training towards integrated programs where this training is heavily complemented by action-based learning. In this way, the current study indicates that athlete leadership development in professional teams is moving towards leadership development recommendations presented in contemporary organisational research [50, 54–58]. The classroom based ‘*learn-to lead*’ type programs described by participants in our study provided important foundational leadership knowledge (e.g., understanding diverse leadership styles, individual leadership preferences and leadership as a relationship-based influence process) and developed players’ understandings of self. Although these conceptual understandings of leadership were central to athlete leaders learning how to leverage their leadership influence it was clear that this knowledge alone did not adequately prepare them for their ALG role. Specifically, it was evident the most impactful ALG development programs integrated contemporary leadership theory with well-designed experiential and social learning opportunities underpinned by guided reflective practice.

Findings from the current study highlight the central role of experiential learning in the development of players for ALG roles in professional teams [53]. Although they acknowledged that some experiential learning occurred organically, participants emphasised that experiential learning in this context was most impactful when it was deliberately designed to meet the specific development needs of individual athlete leaders; involved authentic leadership tasks (e.g. conducting a team meeting, presenting part of a game review, providing feedback to a teammate, pubic speaking); and was scaffolded in a way that ensured the developing athlete leader was ‘stretched’ but felt adequately prepared and supported. It is noteworthy that our results highlight the fine balance that exists between a ‘challenge’ or a ‘threat’ response for a developing athlete leader. Given this, it is critical that these situated experiential learning experiences are carefully aligned with the readiness of individual athlete leaders and undertaken with the support and feedback of expert facilitators in an environment in which the developing leader feels psychologically safe [59].

It was evident that the experiential learning of athlete leaders in these professional teams was often augmented by social learning opportunities (both deliberate and organic) with each other and other high-performance leaders. These included, for example, brainstorming within the ALG, leadership stories from guest speakers, mentoring, and structured opportunities to network with and learn from other HP leaders. This type of mutual sharing has been previously identified as an effective approach in leadership and sports psychology interventions and as a useful tool to facilitate personal development [19, 58]. Our results, however, highlight that the impact of deliberate social learning opportunities in professional teams was often diminished due to a lack of planning. When deliberate social learning opportunities were insufficiently ‘engineered’, for example, when the ‘peers’ (i.e., guest speaker, mentor etc.) lacked credibility, genuine understanding of high performance or the right motivation, these social learning opportunities risked being perceived as a waste of time and potentially undermined athlete leaders’ engagement in the process of leadership development. Consequently, for social learning to really add value to the development of leaders in this unique environment the concept of ‘fit’ appears critical.

Participants in the current study believed that guided reflective practice played a central role in the development of players for ALGs in these professional teams. Guided reflective practice was seen as an important tool to optimise learning from athlete leadership development activities (i.e., leadership theory, experiential and social learning opportunities). Although the role of reflective practice in athlete leadership development has been previously identified [19], participants in the current study emphasised the importance of the reflective process being ‘guided’ by an experienced facilitator. They believed that guiding/facilitating the reflective process helped athlete leaders to unpack their leadership experiences more effectively, and explore different perspectives, interpretations, and alternative leadership practices. In this way, guided reflective practice ‘tied’ the various elements of a leadership development program together and made it meaningful. Previous athlete leadership development research has pointed to the importance of creating an environment to support open reflection [19]. Day (2000) suggested that the effectiveness of any leadership development is contingent on the participants willingness to accept feedback and be open to changing their behaviour [55]. Our results indicate that the professional football environment may be somewhat unique in this way. As a result of the work that has been done to develop a high trust environment where robust feedback is normalised, players in these professional teams appear to have an increased threshold for being challenged with honest feedback, so long as they perceive it be constructive to their performance. Findings from the current study suggest the receptiveness and openness that professional players demonstrate may provide facilitators greater scope to effect behavioural change through guided reflective practice because they can potentially ‘challenge’ these players to reflect to a level that leaders in other contexts may not be comfortable with. This may in turn may afford facilitators in professional sport, a unique opportunity to accelerate leadership development.

### Practical implications

As presented here, the findings offer something of a road map for coaches, teams, and leadership facilitators to guide the design and implementation of leadership development programs for ALGs in professional sports teams.

- Given the findings, it would seem that the implication for those responsible for overall program management and resource allocation in professional sport teams using ALGS is to prioritise athlete leadership development and invest in the services of an experienced leadership facilitator to lead the development of players for ALG roles.
- Leadership facilitators should consider using personality assessments (e.g., DISC, Myers-Briggs) and 360 type feedback tools to enhance developing athlete leader’s self-awareness and understanding of their teammates.
- Coaches are advised to be mindful of providing meaningful opportunities within each training week for developing athlete leaders to practice their leadership skills. This requires coaches to be intentional about athlete leadership development, be willing to delegate and to invest the time to plan these opportunities.
- Leadership facilitators and coaches should work collaboratively, along with developing athlete leaders, to identify and design ‘on-the-job’ assignments that provide athlete leaders with opportunities to practice their leadership skills in the specific areas where this will have the most impact. Care should be taken to ensure these leadership ‘tasks’ are sufficiently challenging to ‘stretch’ developing athlete leaders, but not so difficult that they may feel overwhelmed and lose confidence in their leadership capability.
- The provision of opportunities to learn from other HP leaders should be carefully considered. Coaches and leadership facilitators should do their homework before bringing in guest speakers, mentors etc. to ensure these individuals have the necessary understanding of high- performance environments to be credible and are the right ‘fit’ for the team/individual they will be engaging with. It is also important that these types of opportunities are part of a strategic athlete leadership development program and have a clear purpose rather than simply haphazard, opportunistic activities undertaken ‘for the sake of it’.
- To leverage the benefits of guided reflective practice that participants in the current study described, teams require the expertise of an experienced leadership facilitator and a high trust environment.
- Those responsible for designing athlete leadership development programmes in professional teams should consider how to integrate guided reflective practice throughout all elements of the program.

Further, our findings suggest those responsible for athlete leadership development in professional sports teams should consider how best to develop holistic and personalised leadership development initiatives that bring together the various elements (i.e., foundational leadership knowledge, structured opportunities to learn leadership from other experienced leaders and to practice developing leadership skills in authentic situations) that participants in this study identified as beneficial for developing ALG leaders.

### Strengths, limitations, and directions for future research

In our opinion a notable strength of the current study is the nature of the sample. Professional head coaches and players from athlete leadership groups at this level represent an under-researched, difficult to access, elite cohort [60, 61]. A sample of 30 participants from 17 football clubs across four professional leagues is relatively sizeable compared to other studies conducted in this context and the broad representation provides important breadth. The inclusion of both head coach and athlete leaders’ perspectives is also a unique strength of the current study, in contrast to much of the existing research in this area that has focused on either coach or athlete leaders’ perceptions of leadership development programs. In addition, we believe the depth of participant responses is a key strength of the study. As discussed within the results, participants in this environment demonstrated a propensity to connect with and trust individuals they perceived to have a genuine understanding of high-performance environments. This was reflected within the interview process. As a result of the first author sharing her background in elite sport and establishing a strong rapport in communications preceding the interview, participants perceived a level of shared understanding of the high-performance sport environment which resulted in participants openly sharing their experiences providing in-depth insights they may be less likely to share with a perceived ‘outsider’, such as an academic without high-performance sport experience. Taken together the strength of the sample and the quality of the data contribute to findings that offer a unique insight into an underexplored area of research. It is, nevertheless, important to acknowledge the limitations of this study. Firstly, the findings represent the perceptions of head coaches and players from ALGs only, the perceptions of other team members are not included. Secondly, the effectiveness of the various leadership development opportunities was not evaluated. Although this study sheds light on the leadership development that coaches and athlete leader’s themselves perceived to be beneficial for ALG roles it may be the case that the effectiveness of these leadership development initiatives can only be fully understood by examining the outcomes for the teams and team members in receipt of this leadership. Future studies may look to quantitative methods to evaluate the effectiveness of the various leadership development practices and thereby provide further evidence to inform the implementation of leadership development offerings in this context. It may also be worthwhile to explore the perceptions of other team members and leadership facilitators to establish a more global understanding of athlete leadership development in this professional sport environment.

## Conclusions

This study provides coach and athlete leader’s perceptions of the development opportunities that have been afforded to players to prepare them for their role in an ALG. The study provides important insights that may assist coaches, teams, and leadership facilitators with the design and implementation of athlete leadership development programs within professional teams. Results illustrate that athlete leaders benefit from developing enhanced understanding of leadership as a multidimensional relational concept; recognising diverse leadership styles and preferences; and gaining insight into their personal influence. However, it is evident this theoretical knowledge alone is insufficient to equip players for a professional team ALG role. Players need opportunities to practice their developing leadership skills in authentic and appropriately challenging situations with support and facilitation. Findings point to the importance of systematic, individually tailored leadership development that includes scaffolded, structured experiential learning and meaningful interactions with other successful high-performance leaders. Further, this study reinforces the value of guided reflective practice in leadership development, highlighting how this process can enhance learning and transfer from leadership development initiatives [19]. Finally, this study adds weight to calls from other researchers for coaches to be consistently intentional in employing athlete leadership development strategies [9, 10, 19, 21, 22].

## Supporting information

S1 File Interview guide. (DOCX)

S2 File Quotes exemplifying understanding leadership. (DOCX)

## References

[1] Haddad G, O’Connor D, Burns K. The decision to adopt a formal athlete leadership group: Qualitative insights from professional football coaches. Psychology of Sport and Exercise. 2021; 52: 101803.

[2] Duguay AM, Hoffmann MD, Guerrero MD, Loughead TM. An examination of the temporal nature of shared athlete leadership: A longitudinal case study of a competitive youth male ice hockey team. International Journal of Sport and Exercise Psychology. 2020; 18: 672–686.

[3] Duguay AM, Loughead TM, Hoffmann MD, Caron JG. Facilitating the development of shared athlete leadership: Insights from intercollegiate coaches. Journal of Applied Sport Psychology. 2020; 8: 1–22.

[4] Fransen K, Vanbeselaere N, De Cuyper B, Vande Broek G, Boen F. The myth of the team captain as principal leader: Extending the athlete leadership classification within sport teams. Journal of Sports Sciences. 2014; 32: 1389–1397. doi: 10.1080/02640414.2014.891291 24660668

[5] Johnson T, Martin AJ, et al. Collective leadership: A case study of the All Blacks. APMBA (Asia Pacific Management and Business Application). 2012; 1: 53–67.

[6] Fransen K, Haslam SA, Steffens NK, Peters K, Mallett CJ, Mertens N, et al. All for us and us for all: Introducing the 5R shared leadership program. Psychology of Sport and Exercise. 2020; 51: 101762.

[7] Mertens N, Boen F, Steffens NK, Cotterill ST, Haslam SA, Fransen K. Leading together towards a stronger ‘us’: An experimental test of the effectiveness of the 5R Shared Leadership Program (5RS) in basketball teams. Journal of Science and Medicine in Sport. 2020; 23: 770–775. doi: 10.1016/j.jsams.2020.01.010 32107173

[8] Blanton JE, Linhart C, Ault KJ. Flipping the script: Using an online captain’s course to leverage leadership development training with youth club soccer athletes. Journal of Sport Psychology in Action. 2019; 10: 151–9.

[9] Cotterill ST, Cheetham R. The experience of captaincy in professional sport: The case of elite professional rugby. European Journal of Sport Science. 2017; 17: 215–221. doi: 10.1080/17461391.2016.1245788 27802785

[10] Gould D, Voelker DK, Griffes K. Best coaching practices for developing team captains. Sport Psychology. 2013; 27: 13–26.

[11] Bolden R, Jones S, Davis H, Gentle P. Developing and sustaining shared leadership in higher education. 2015 Project Report. London: Leadership Foundation for Higher Education.

[12] Carson JB, Tesluk PE, Marrone JA. Shared leadership in teams: An investigation of antecedent conditions and performance. Academy of Management Journal 2007; 50: 1217–1234.

[13] Burke CS, Stagl KC, Klein C, Goodwin GF, Salas E, Halpin SM. What type of leadership behaviors are functional in teams? A meta-analysis. The leadership quarterly. 2006; 17: 288–307

[14] Drescher MA, Korsgaard MA, Welpe IM, Picot A, Wigand RT. The dynamics of shared leadership: Building trust and enhancing performance. Journal of Applied Psychology. 2014; 99: 771–783. doi: 10.1037/a0036474 24731178

[15] Fausing MS, Joensson TS, Lewandowski J, Bligh M. Antecedents of shared leadership: empowering leadership and interdependence. Leadership and Organisational Development Journal. 2015: 36: 271–291.

[16] Kezar AJ, Holcombe EM. Shared leadership in higher education. Washington, DC: American Council on Education. 2017.

[17] Jones R, Standage M. First among equals: shared leadership in the coaching context. In: Jones RL, editor. The sports coach as educator: Re-conceptualising sports coaching. London: Routledge; 2006. pp. 65–76.

[18] Fransen K, Vansteenkiste M, Vande Broek G, Boen F. The competence-supportive and competence-thwarting role of athlete leaders: an experimental test in a soccer context. PloS one. 2018; 13: e0200480. doi: 10.1371/journal.pone.0200480 29995935 PMC6040764

[19] Cotterill S. Developing leadership skills in sport: A case study of elite cricketers. Case Studies in Sport and Exercise Psychology. 2017; 1: 16–25.

[20] Fransen K, Vanbeselaere N, De Cuyper B, Coffee P, Slater MJ, Boen F. The impact of athlete leaders on team members’ team outcome confidence: A test of mediation by team identification and collective efficacy. The Sport Psychologist. 2014; 28: 347–60.

[21] Cotterill ST, Fransen K. Athlete leadership in sport teams: Current understanding and future directions. International Review of Sport and Exercise Psychology. 2016; 9: 116–33

[22] Voelker DK, Martin EM, Blanton JE, Gould D. Views and practices of high school coaches on the education and training of team captains in leadership. Journal of Leadership Education. 2019;18.

[23] Voelker DK, Gould D, Crawford MJ. Understanding the experience of high school sport captains. The Sport Psychologist. 2011; 25: 47–66.

[24] Voight M. A leadership development intervention program: A case study with two elite teams. The Sport Psychologist. 2012; 26: 604–623.

[25] Duguay AM, Loughead T, Munroe-Chandler KJ. The development, implementation, and evaluation of an athlete leadership development program with female varsity athletes. The Sport Psychologist. 2016; 30:154–166.

[26] Navarro K, Malvaso S. Toward an understanding of best practices in student-athlete leadership development programs: Student-athlete perceptions and gender differences. Journal of Applied Sport Management. 2015; 7: 23–43.

[27] Bucci J, Bloom GA, Loughead TM, Caron JG. Ice hockey coaches’ perceptions of athlete leadership. Journal of Applied Sport Psychology. 2012; 24: 243–259.

[28] Levitt HM, Motulsky SL, Wertz FJ, et al. Recommendations for designing and reviewing qualitative research in psychology: Promoting methodological integrity. Qualitative psychology. 2017; 4:1:2.

[29] Adams WC. Conducting semi-structured interviews. In; Hatry HP, Newcomer KE, Wholey JS, editors. Handbook of practical program evaluation. John Wiley and Sons Inc; 2015. pp. 492–505.

[30] Jones I, Gratton C. Research Methods for Sports Studies: 3rd ed. Taylor and Francis Group; 2015.

[31] Smith BM, Sparkes AC. Routledge handbook of qualitative research in sport and exercise. London: Routledge; 2016.

[32] Smith B, McGannon KR. Developing rigor in qualitative research: Problems and opportunities within sport and exercise psychology. International Review of Sport and Exercise Psychology. 2018; 11: 101–121.

[33] Braun V, Clarke V. Conceptual and design thinking for thematic analysis. Qualitative Psychology. 2022; 9: 3–26.

[34] Aziz M A, Kenford S. Comparability of telephone and face-to-face interviews in assessing patients with posttraumatic stress disorder. *Journal of Psychiatric Practice*. 2004; 10: 307–313. doi: 10.1097/00131746-200409000-00004 15361745

[35] Haslerig SJ. Phone it in: Reflections on the use of phone interview methods with college athletes. Journal for the Study of Sports and Athletes in Education. 2021; 15: 171–191.

[36] Braun V, Clarke V, Weate P. Using thematic analysis in sport and exercise research. In: Smith B, Sparkes AC, editors. Routledge handbook of qualitative research in sport and exercise. London: Taylor and Francis; 2016. pp. 191–205.

[37] Braun V and Clarke V. Reflecting on reflexive thematic analysis. Qualitative research in sport, exercise and health. 2019; 11: 589–597.

[38] Terry G, Hayfield N. Reflexive thematic analysis. In: Handbook of qualitative research in education. Edward Elgar Publishing. 2020: 430–441.

[39] Martindale RJ, Mortimer P. Talent development environments: Key considerations for effective practice. In Collins D, Button A, Richards H, editors. Performance psychology: A practitioner’s guide. Elsevier Limited; 2011: 65–84.

[40] Pedler M. Developing within the organisation: Experiences with management self-development groups. Management Education and Development. 1986; 17: 5–21.

[41] Showry M and Manasa KV. Self-Awareness-Key to Effective Leadership. IUP Journal of Soft Skills. 2014; 8: 15–26.

[42] Thach EC. The impact of executive coaching and 360 feedback on leadership effectiveness. Leadership and Organisation Development Journal. 2002; 23: 205–214.

[43] Luthans F, Peterson SJ. 360‐degree feedback with systematic coaching: Empirical analysis suggests a winning combination. Human Resource Management: Published in Cooperation with the School of Business Administration, The University of Michigan and in alliance with the Society of Human Resources Management 2003; 42: 243–56.

[44] Amagoh F. Leadership development and leadership effectiveness. Management Decision. 2009; 47: 989–999.

[45] Chelladurai P. Leadership in sports: A review. International Journal of Sport Psychology. 1990; 21: 328–354.

[46] Høigaard R, Jones GW, Peters DM. Preferred coach leadership behaviour in elite soccer in relation to success and failure. International Journal of Sports Science and Coaching. 2008; 3: 241–50.

[47] van Dalfsen G, Van Hoecke J, Westerbeek H, De Bosscher V. Coaches’ views on how to develop shared leadership. Sport, Business and Management: an international journal. 2021; 11: 265–286.

[48] Price MS, Weiss MR. Peer leadership in sport: Relationships among personal characteristics, leader behaviours, and team outcomes. Journal of Applied Sport Psychology. 2011; 23: 49–64.

[49] Kolb DA. Experiential learning: Experience as the source of learning and development. 2nd ed. New Jersey: FT Press; 2014.

[50] Zenger JH, Folkman J. Developing leaders- Define what leadership means to you. Executive Excellence. 2003; 20: 5.

[51] Crenshaw JT. Use of video-feedback, reflection, and interactive analysis to improve nurse leadership practices. Nursing administration quarterly. 2012; 36: 260–267. doi: 10.1097/NAQ.0b013e318258c4e0 22677967

[52] Fulmer RM, Conger JA. Developing leaders with 2020 vision: companies can develop deep, enduring bench strength by combining succession planning and leadership development to create a long-term process for managing the talent roster. Financial Executive. 2004; 20: 38–42.

[53] Kolb DA, Boyatzis RE, Mainemelis C. Experiential learning theory: Previous research and new directions. In: Sternberg R, Zhang L, editors. Perspectives on thinking, learning, and cognitive styles. Routledge; 2001. pp. 227–248.

[54] Bennett B. Job rotation: Its role in promoting learning in organizations. Development and learning in organisations. 2003; 17: 7–9.

[55] Day DV. Leadership development: A review in context. The Leadership Quarterly 2000;11: 581–613.

[56] Leskiw SL, Singh P. Leadership development: Learning from best practices. Leadership and Organisation Development Journal. 2007; 28: 444–464.

[57] Denston I, Gray J. Leadership development and reflection: What is the connection? International Journal of Educational Management. 2001; 15: 119–124.

[58] Mann D. The missing link: Lean leadership. Frontiers of Health Services Management. 2009; 26: 15–26. 19791484

[59] DeRue DS, Wellman N. Developing leaders via experience: the role of developmental challenge, learning orientation, and feedback availability. Journal of Applied Psychology. 2009; 94: 859–875. doi: 10.1037/a0015317 19594230

[60] Fransen K, Haslam SA, Mallett CJ, Steffens NK, Peters K, Boen F. Is perceived athlete leadership quality related to team effectiveness? A comparison of three professional sports teams. Journal of science and medicine in sport. 2017; 20: 800–806. doi: 10.1016/j.jsams.2016.11.024 28214098

[61] Leo FM, García-Calvo T, González-Ponce I, Pulido JJ, Fransen K. How many leaders does it take to lead a sports team? The relationship between the number of leaders and the effectiveness of professional sports teams. PLoS One. 2019; 14: e0218167. doi: 10.1371/journal.pone.0218167 31181130 PMC6557507

