# Supplementary material for: Developing players for athlete leadership groups in professional football teams: Qualitative insights from head coaches and athlete leaders
Source: PLoS One. 2022 Aug 3;17(8):e0271093. doi: 10.1371/journal.pone.0271093 (PMC9348637; doi:10.1371/journal.pone.0271093)
Supplement: S1 File — (DOCX) [file pone.0271093.s001.docx]

**Interview Guide**

Coach version:

Section 1 Background

1. Can leadership skills be developed in your players or are these inherent?
2. On what basis are players selected into formal leadership roles?
   1. What is the purpose of the leadership group
   2. How does the AL group ‘work’ within your team and with you?
   3. What would be the most enjoyable/satisfying aspects for a player being in the leadership group?
   4. What would be the most challenging aspects for a player being in the leadership group? Why?

Section 2 Leadership development

1. Can you describe the leadership development that players receive for their ALG role? Do you bring in an external consultant or another staff member to assist with this?
2. Is this provided prior to their appointment to the leadership group? If so, at what stage? I.e. after identification or actual selection? Or is this only provided once they have been appointed
3. How well do you think this prepares players for those leadership roles and why?
4. Which leadership development activities work best and why do you think this is?
5. What aspects of their leadership role do you think athlete’s find most challenging? Why?
6. What additional training and/or development opportunities do you think would benefit players transitioning to a leadership role?

Section 3 Closing comments

1. Would you like to add anything else related to our interview?
2. Do you have any final questions or comments?

Athlete version:

Section 1 Background

1. What is the purpose of the leadership group; and what specifically is your role?
2. Why do you think you were selected for a leadership role within your team?

Section 2 Leadership development

1. What skills, knowledge, attributes do you bring to this role?
2. How does the AL group ‘work’ within your team?
3. What are the most enjoyable/satisfying aspects of being in the leadership group? Why?
4. What are the most challenging aspects of being in the leadership group? Why?
5. What aspects of the role are different to your perceptions before joining the leadership group?
6. How well-prepared did you feel when first appointed to a leadership role in the team?
7. Are there leadership experiences from other areas of your life that you believe may have helped prepare you for your current leadership role (i.e. at school, work home or in other teams etc.) can you describe these and how you think they have helped to develop your leadership skills?
8. What training or development did you receive in preparation for your role in the ALG?

- Was this training individualised or did the group receive the same training?
- Who delivered this training? When and where was it delivered and what was the duration of the training?
- Did you feel this training equipped you well for your leadership role? If yes, why, If no why not?

1. Did you receive training or support during your time in the leadership group? Can you describe this support/training? Was this helpful? One-off or ongoing support/training?
2. In your experience, what leadership development was the most helpful and why?.
3. Is there any specific additional training which you feel may help you to fulfil your leadership role more effectively?
4. What advice would you give to next year’s leadership group?

Section 3 Closing comments

1. Would you like to add anything else related to our interview?
2. Do you have any final questions or comments?
