# Supplementary material for: Developing players for athlete leadership groups in professional football teams: Qualitative insights from head coaches and athlete leaders
Source: PLoS One. 2022 Aug 3;17(8):e0271093. doi: 10.1371/journal.pone.0271093 (PMC9348637; doi:10.1371/journal.pone.0271093)
Supplement: S2 File — (DOCX) [file pone.0271093.s002.docx]

**Quotes exemplifying Understanding Leadership**

| I think everyone sort of starts out being thrown in there as a leader just because you perform on the field. Seeing what we do now, the [leadership] development, I think back, and I had no idea…It's come a long way. There was never really any massive focus. When I started it was like, "Oh, you're in the leadership group," but we wouldn't even catch up much, there was no massive focus like there is now, how much more in depth the leadership side is (P9) |
| --- |
| Players naturally fall into leadership positions because they’ve been there a long time but maybe they haven’t ever been taught what it actually means or what type of person they actually are or what type of leader they are. So that’s definitely one thing that you could help people do is understand more about themselves so then they’ve got more self-awareness in terms of being able to know their effect or their impact on players around them. You don’t know what you don’t know so you could actually invest in people so then they can discover more about themselves. (P3) |
| Most of that [leadership development] work is done in pre-season, when we can, when we've got a bit more time, because we're not worrying about going out and winning. (C6) |
| I think it’s helping them not worry about what other people think. Last year, when we started [as an ALG] we would have been embarrassed to pick each other up on standards. (P2) |
| Sometimes it’s just even the senior leadership guys spending time with those younger guys, taking them out to dinner, developing relationships (C3) |
| They need to develop the ability to deal with everyone. All teammates, no matter how rough around the edges they are, what school they'd come from, what family background they had. The next thing I think those guys [the ALG] need is to build relationships with all players and coaches. (C7) |
| When you’re new to the leadership group, you need to understand different personalities of players and different traits of leadership, whether you lead by example or if you're the type that wants to rant and rave on. You've got to get to know yourself and know what your group responds to. (P13) |
| Player leaders that have come through an organisation in a professional sense, a professional sporting sense, they're highly skilled in delivering and receiving feedback, and what drives high performance, and relationships. (C8) |
| Understanding that you need to get into the world of all these players [teammates], building relationships is pivotal, so you can have a really important effect. Then I guess, when you're having these conversations, like a bit of small talk is necessary, but then you go, "Hey, how are you going and they go, no, no all good, you know, they just have their manly front up, and everything's great and everything good, and that's where I tend to share my vulnerability piece of, you know I went through XYZ and they go, oh, and they can really respond to that. I find asking again really, what's going on, How are you really doing?” Then you find out some good feedback. (P10) |
| I’ve learnt you need to invest in building relationships... to be honest with you I take a long time to sort of form them. Making sure they're real as well and you actually give them the time that enables you to, when you need to have a tough or serious conversation you can ...... and knowing it's coming from a place where it's valid rather than just for the sake of it or you know not liking someone, being able to say I'm doing this for the sake of you getting better at ... rather than just explaining to someone who you don't really know, without really forming a relationship, and their thinking it's coming from a different place. (P11) |
| I reckon I wasn't prepared at all. It kind of went on experience …and I was just chucked in there as a leader (P8) |
| I’ve come to understand that you need to build relationships with them, get to know them, you realize how much they've got going on in their home life. For example, with the Polynesian players, A lot of them are supporting their whole family. You know, extended families and all; they’re sleeping on the floor in the garage, and they might be struggling on the field. So then if you're coming down on them extra hard here, they just crumble because they got so much pressure coming down on them. I think I learnt, you need to build that relationship with them show them you care, then it is a little bit easier to be a little bit hard on them sometimes, but xxx[the facilitator] has always taught us, "Always finish with a positive." I know you can do this. You've done it before” and you're supportive of them. (P9). |
| In the early days I used to find it really tough but I've just learned to get over that and come back to the values. I was taught that you challenge the behaviour, you support the person. So, I always acknowledge that, even just as a repetition thing to say, "Hey you're a great bloke, I'll catch up for a coffee or beer with you no problem, but your behaviour needs to improve here. So a one to one I can do that with no problem because I defer to our values, and just say like, "Hey, you agreed to this", type of thing. "If you want to get selected", It comes back to incentivizing them. Really like, it's not about me having a go at you, it's about getting you up, type thing. (P10) |
| We did this thing, so last year, just to get to know each other a bit more we'd sort of get up in front of the group and talk about yourself growing up. And so you'd have these three photos and you'd talk about ... so a lot of them were really emotional stories or things that people have gone through that would give you a different perspective on them. It was pretty emotional. So you'd learn these things about your teammates that you wouldn't know and would sort of piece together maybe why they are like they are. making yourself vulnerable. It definitely brought us a lot closer. Now when I think about my teammates I sort naturally think about what they have been through which really suits my sort of leadership style. I think I probably think about it more maybe than other people. So that helps me with that sort of caring and supporting leadership. (P5) |
| I kind of just understood that not all leaders were the same, but my coach as well explained to me, he didn't want me to change. He didn't want me to do anything different. What he saw in me was enough for him to want me to be a leader, and he wanted me to stay the same and not feel any added pressure to change the person that I was, so I was happy to do it my way. (P12) |
| My leadership style is isn't someone who challenges really strongly, or steps on toes. My strength is probably my communication and stuff on game day and I'm pretty empathetic. I'm pretty caring and understanding so I think that's why my peers voted me, because they obviously feel comfortable around me. The other guys in the leadership group have different styles, two are more challenging, and the other is more strategic, we all bring something different. (P5). |
| He's [Senior ALG player] excellent – doesn't mind being unpopular. He will hold any person to account and is very strong in that regard. The area that he wants to develop is that relationship side, so he's very good at holding them to account, but often that can come across as intimidating to the younger players. But he is aware of that and understands he has to grow some more tools in his tool box to deal with the relationship side of things– so that's one quality he’s working on developing (C10) |
| Learning about millennials, you know the use of their phone and then how all these factors throughout their life is really making it tough for them once they get into the workforce. I recognised that’s our demographic, you’ve got a lot of kids coming straight into the xxxx [team] after school, so everything up until then has been like a pat on the back, “you’re killing it”, they haven’t even had to leave mum and dad’s house then they’re in here to a high performance environment and all of a sudden you're not getting picked for the team, training’s hard, someone’s just yelled at you on the footy field and instead of you being the hero every week and then you go back to your phone and you look through it and then all these things like, you're not getting that instant gratification that your whole life has been built up towards that point. So, all of a sudden, kids then go, well, I suck, I’m terrible at everything, it’s never going to happen, I’m never going to make it. It just made so much sense and helped me understand the different approach I need myself towards this younger group (P1) |
| I was facilitating with our leadership facilitator, this discussion, pre-season with the senior leadership – we worked with them around how they could have an influence on the rest of the group, how they get the boys [teammates] on board (C6) |
| It's definitely a lack of confidence, definitely a lack of comfort in speaking in front of groups and having the clear thought processes to communicate what they're thinking. And then the other part is the performance issue, where we're at as a team, and where they’re at as an individual and player, I mean these guys were always the best players in every team they played in. And everyone looked to them so they lead by example but then when the pressures is on them, when they're the ones not playing well, they do withdraw into themselves a little bit and try to get their own house in order. And that means that they’re thinking, I'm not going to think about that guy and that guy for the next few weeks because I’ve got to get myself right. And I think that's human nature, but the best leaders are able to deal with both of those things. (C2) |
| Just understanding how much you can actually do for someone and how leadership's not all about just barking orders. It's about building relationships and then it's almost coming from the point of care. (P4) |
| It was definitely like a shift for me because my personality is the jokey, having a good time, take the mickey out of situations but then you know when we’re on we’re on, so that’s my personality. And then when I went into the leadership group I started to get worried that I had to tighten that up so I couldn’t really be the jokey, have a good time person and I had to tighten that all right up. But learning from last year to this year, people don’t want your personality to change. As long as you're not doing the opposite about what you're trying to get people to do then you're allowed to be yourself - so many people have different personalities. the reason why you are where you are is because you were yourself so why change there? They don’t want you to tone down your aggression out on the field, they don’t want you to tone down your competitiveness and they want you to keep doing that. But they also want you to be that voice and that excitement around the group so that’s something that I had to learn as well. I started trying to be real proper and be like no, we shouldn’t joke about that and the guys were like who is this bloke? I didn’t know that I had such an ability to influence the group and change the culture , You know, the ability to shape the group into the right direction and that I could still be myself (P2) |
| I’d never seen myself as a leader, I'd always seen myself as bit more of a joker within the team, and someone always up for a laugh and just not what I thought was a leader. In that role, you'd expect someone who's serious, someone who when they talk, players listen, someone physically as well with a real presence, and I never really had any of those things, you know? It wasn't until he [facilitator] explained to me that leaders come in all different forms and ways of being he said, but he noticed within me that I did have that presence, just not in the way that I assumed, you know? (P12) |
| It’s learning to understand what people respond to, and you communicate with different people in different ways, if they’re not super confident, you don’t spray them in front of the group, you say a little something on the side. But if they’ve got a dominant personality you can maybe spray them in front of the group and they’ll take that really well so little things like that. (P1) |
| A lot of informal training. That can't be underestimated. Because you're involved in discussions at a whole different level. Both in a performance sense but also in an off-field sense. You're a lot more aware of what goes on around the club - the impact that different things have on the entire organisation. Members of the leadership group become much more aware of things that impact performance other than just playing. (C8) |
| He [leadership facilitator] did this thing, this survey, where we critiqued each other, on our leadership, so like strengths and weaknesses, and understanding our leadership style, we just had a really good, honest conversation around it, it was really helpful. It really brought it to light for me that you’ve actually got to deal with people in different ways, if you're going to give someone individual feedback, you got to go around different ways if you want the best outcome, the best result from it. (P9) |
| We've got a regular weekly leadership meeting … just the leaders together with xxx [facilitator], which is just learning and development stuff for us. At the moment, actually, we're reading this book about the growth mindset. He [facilitator] researches what he thinks is good, that can relate to us, and then he delivers it to us, and we just have a conversation around it. (P8) |

**Quotes exemplifying Experiential learning**

| I haven't really had any formal [leadership] development, it's been more just yeah, learning as you go. (P13) |
| --- |
| For me, it was definitely yeah, learning as I went along…learning on the job. (P12) |
| In our program the leadership development has about 15% formal leadership activities, training, call it what you will, and 75% of it is experiential learning, on the job learning with me or the facilitator giving them [the ALG] feedback and having the conversations. There's another group, our emerging leader’s group and we have another facilitator, it's one of our coaches that runs that group. He's a person that has been a leader in his own time as an athlete, and those emerging leaders run in almost exactly the same program, but it's tailored more for them and the role they'll play. They'll be given some roles within our organization, so they'll also be responsible for certain facets of our program, at a slightly lower level. (C6) |
| The most meaningful learning is through experience. For example, having those losses and then learning from them, and being better for the next time., having a big loss and trying to get a group out of a tough situation; losing the game, looking back, getting the learning from that, and being better when you're in those situations again, if that makes sense. (P8) |
| They [developing leaders] become more comfortable to be able to talk to that group. I always say, everything's like a habit. If you practice talking in front of the group, you get better at it. If you don't practice it, you don't do it. I think being able to create that environment where they have an opportunity to practice their leadership skills is so important (C3) |
| In my younger days as a leader, if we’d had a really tough loss I would have just been filthy for ages, but you learn. I've learnt that as a leader, you can't do that, you need to get over it quick because it's not going to help anyone. You got to try and portray positivity to the group. You know, instil confidence in them that we're all right. Put that across and build them back up. As much as you want to come down on them really hard, sometimes that's not always the best thing. So you make mistakes along the way but you just learn from them, you learn through experience. (P9) |
| Transition has always been a focus of that leadership group. One of their key priorities is to work with that next group underneath - the emerging leaders - and give them responsibilities, and provide opportunities for them, even though they're not in the leadership group. That’s an important part of the leadership group responsibility. The leadership group have discussed what they see as the next group of leaders and have worked to put a plan in place to develop that next group. (C8) |
| Let them deliver it, and work on their delivery, and work on their confidence in that situation, catch up with them and encourage them, before meetings, to have input, and give them confidence, give them some formal responsibilities, as in monitoring some of the off-field responsibilities that go with being a footballer, such as making sure we’re on time, and just doing different things (C4) |
| I think you need to take a progressive approach developing those leaders, in that first year, ensuring that the conversations that have to be led by the leadership group with the team and with other players are not life and death conversations. You know, it’s just a little bit like “we’d just like to see you do a little bit more of this and a little bit more of that”. Whereas in year five it's, "if we're not happy with the way you're playing, we don't think you training hard enough, if you don't act together, we're going to suggest to the coach that you're not on the team anymore." You know they're pretty tough conversations, they need to build up to that. (C2) |
| The next generation of leaders need to be fostered, leadership group will come up with various responsibilities, genuine leadership responsibilities, for that next group to be able to work on their leadership skills, and start to develop them, because they see that as a critical part of their role, to develop the next group of leaders. (C8) |
| The thing I’ve really struggled with, because I like to be like, is having to give that hard feedback, I don’t like confrontation…I’ll avoid it…but it’s got easier over time and with help from xxxx [facilitator] on how to go about it. (P4) |
| Underneath the leadership group there are three or four really natural young leaders that we can see, but we don't want to burden yet. We just want to let them be them and build a career and take some responsibility for their part of the ground that they most often play in and gradually increase those responsibilities. (C7) |
| With the players in leadership groups, they need to be given time to just be them and find their feet. (C16) |
| We've also got an emerging leadership group, which is these young players, as well, underneath the senior leadership group. These are our young emerging leaders who only meet every couple of weeks, they're a little bit more down at a lower level, working away at their leadership craft (C9) |
| I'd always have three tier leadership, with the senior guys, the next generation and then your rookies, but integrated throughout. (C3) |
| They have an emerging leadership coach, it’s another really good way to engage other players in a group to prepare them for future leadership. And some of those guys have come through and are now part of the leadership group. We'd also have an annual emerging leaders camp, so those five guys would go away with the leadership coach and the head coach and spend two nights together get to know each other a bit better, try to get some connection. And it wasn't so much the formal activity they did while they're away. It's more just that connection, that cohesion that they formed and starting to see themselves as leaders (C5) |
| I give them [the athlete leaders] two or three emerging leaders to mentor. Which gives them some responsibility and they then are responsible for identifying some leadership opportunities for a couple of the young guys coming through. (C1) |
| Emerging leaders groups, which is guys outside of the senior leadership group will be part of that group and they will actually be facilitated by an external person, who will meet with them as a group weekly, and individually throughout the week…to guide them through the process of leadership development. What he does is he meets every Monday morning, he has breakfast with that group, they review the game together and their contributions in the game and they appoint somebody who would need to go back and then present that, their collective thoughts, back to an assistant coach, before the team meeting. (C11) |
| I came from the emerging group. It had xxxx [facilitator]who comes in once every two weeks, with like a leadership program, working with the whole team and leading an emerging generation leader’s group. So, I had that support early on. (P6) |
| We did a lot of team building type activities, you know, the SA style stuff where they [the ALG] were in command we did quite a number over the last two years because I was cognisant that we had to grow in our leadership capacity. (C10) |
| We’ve to tried to find people’s strengths and work on them in that area or even weaknesses. You’re developing a leadership style, but not too much too soon there needs to be a bit more of a gradual transition. So you're sort of building capacity, letting them practice in the areas they feel comfortable with first. (P2) |
| Because with that democratic system you're voted in as a leader so, theoretically, you're the most qualified to do the job. The reality is you're far from it, so you need to actually be growing and learning and being taught certain things on the run, if you know what I mean. It would have been really helpful to have a sort of facilitator from the start, to help us, answer questions, help us run those meetings and be more effective. (P1) |
| They’re [emerging leaders] certainly getting a lot more confident in speaking in front of the group as they get more practice. Even seeing them chip blokes in the squad who they wouldn’t even really talk to is – not really chip, just trying to get the best out of them, challenging them it’s great. (P2) |
| Conducting court with a group of seven or eight players can be quite nerve-wracking because everyone is looking at you. It's like a teacher on their first day. You can study all you like, but when you get in front of a crowd, it's a hell of a lot different. You’ve got to build their confidence. We put them in as many of those positions as we possibly can. But the planning and preparation is important, in terms of we don't allow people to cowboy up. You just can't let them just wing it. If it's a difficult conversation that needs to be had, I would help them beforehand or go with them. But we’re always looking for opportunities for them to grow as leaders (C9). |
| The coach might ask "Who wants to present this to the group?” He delegates stuff. Having those opportunities, being up in front of the group, speaking publicly etc. and then with practice you can execute, naturally. I was a little nervous speaking in front of the group the first few times, then it becomes ingrained just like anything. I guess opportunity is crucial, I would say preparation plus opportunity equals performance (P10) |

**Quotes exemplifying Learning from others**

| I think the most value I get is from going and watching or listening to people talk about leadership, the most effective way of doing it. (P4) |
| --- |
| Based on what we think is important to build a fully top line leadership group, we want to spend a fair bit of time with senior leaders, exposing them to what a really strong leader looks like. That will often be a person that will come in and talk about why did they lead how they did, like guest speakers. It's not always sport, we do a bit with the military, we do the same with leaders from other footie clubs that have been successful. So they can perhaps pick up some ideas from those people. There are opportunities to ask about how they are able to influence others and support others or challenge others or make others better or get them to come together as a group of people for a common cause, all those sorts of things, we're always looking for those opportunities. (C6) |
| Exposing them [ALG and emerging leaders] to stories of success around leadership and influence and stuff like that is powerful, I think those stories give them a better understanding of leadership. (C13) |
| I've brought in some really good people and influential leaders from other organizations and got them to talk about what it means to be a leader, how to have honest and open conversations in a safe environment. Just talking about how you felt under pressure, it's talking about those sorts of different things, it develops an understanding of leadership. (C3) |
| It’s invaluable, seeing what works for other people. (P1) |
| Observing other leaders, you saw what was effective, what people responded to. (P2) |
| I learnt a lot off those sorts of experienced leaders in terms of how to deal with the changing room and how to approach players and what sort of players respond to different leadership traits. (P13) |
| We went down to xxx [city], to a talk by xxxx [prominent leader] about how he goes about leadership, really listened to that, that was great, then we went to visit xxxx [successful football team from another league], talking to their leadership group about how they go about it, got a lot out of that. We also went to the [headquarters of another football league] after that, sat down with their CEO, talked about how he goes about leadership (P11) |
| You know, with us, as a leadership group, we're trying to go out there and grab some knowledge from everyone, try it out and see what works for us (P8) |
| With Xxxx (mentor) his leadership style is so interesting and just being able to spit ball with him about things, even little things like the reserves are struggling a little bit, we’re trying to get the best out of them like, what can we do in that area, that sort of thing – and he’s a wealth of knowledge in that area. Learning through those conversations is so strong, especially for blokes that aren’t that predisposed to study. Just even chatting and shooting the shit with each other, it’s a way to really bounce ideas off each other and them not having any biasness. They don’t know who these people are personally (P2) |
| One thing they did that worked was they assigned [ALG] players to mentor. The expectation was, to have a weekly catch up. An informal catch up every week. (C1) |
| There are mentors. They have mentors for them [the ALG] and that's everyone. One of them has a mentor from the xxxx foundation, who work with young people, so they're all different, someone to bounce things off (C7) |
| They’ll [ALG players] be assigned a mentor role with one of those rookie young players (C8) |
| Mentoring. Sometimes I have groups of six players underneath each of the leaders and that was their mentor or their go to person. It works both ways, helps them grow as leaders and supports those younger guys too (C3) |
| Having a mentor is crucial. (P10) |
| We did that mentorship thing and it wasn't a very natural sort of setup…But I think if you did find that sort of naturally and had that mentor that you had a really good relationship with and it wasn't sort of forced and awkward, it would be really beneficial. I guess for the guys that have that it would be really good. (P5) |
| I'd be amazed if there is any xxxx [football league] team that had someone externally who has come in and helped to set up a leadership group and train players on leadership. I'd be amazed if any club has done that. I don't think that happens in this sport… I've never experienced that. (C15) |

**Quotes exemplifying Guided Reflective practice:**

| I've seen some facilitators in leadership groups start fights for no reason. It's almost, "We need some confrontation”. Either that or hang on... There's going to be something at play here. But this guy, he would sometimes let sleeping dogs lie if he sensed that was the best way, but if he sensed something that needed to be teased out, he wouldn't let them out of the room until he'd get it out of them. He'd get it out like a splinter. He would find a way and he would look at eyes and look around the room, and if he sensed an opportunity ... And I've always believed, you sit in a lot of meetings and forums and leadership things, and I've always believed that when you feel like you want to get something off your chest and say it, that's the time to say it. And he had a unique gift to look at a player or a person in the room and sense outright that face and he would say, "What do you think? What do you think? And that might be just a start and he would just piece it together. And in the end, they would be adding the pieces for him. He was brilliant, he would tease it out. (C7) |
| --- |
| He would send me a text after every game. And ask me things that came up during the game and about how to deal with it and stuff like that. It allowed me to think about what actually did happen and make me sort of evaluate more how I learn rather than just playing. It made me think about how did you lead? So sometimes if I wasn't having the best game I would probably like write back to him and say I was probably a bit in my own head. I wasn't playing the best game. I had a goal kicked on me and that sort of put me in a funk for 10 minutes. And I probably didn't notice it until now. (P5) |
| It comes down to how well we support those leaders in terms of giving a lot of feedback, asking them to reflect, checking with them all the time. For example, our strongest leader in the leadership group, he's really growing as a leader, and I think he'll develop into a world class leader.… because he's got that reflective practice going. (C10) |
| I think it's very hard to be the best leader you can be if you can't assess yourself, if you don't know who you are and what you are. So self-assessment, self-awareness for me is critical and I think as you grow as a leader, a massive part of leadership is making hard and fast decisions under pressure, in the heat of the moment, you're not going to get them all right. So, when you don't get them right, you have to be big and bold enough to look in the mirror and say, "You know what, I got that one wrong. I'll make sure I don't make the same mistake twice." So, I think that is probably the most powerful training you can have, as a leader is that reflection I mean, there's other things, like speaking in front of a group and public speaking skills and how to carry yourself, body language, all that sort of stuff is so important. But if I had to look at the most critical thing, it would probably be self-assessment and they [ALG] need help to develop that capacity. (C14) |
| We help them to reflect on their performance, we give them feedback on their performance as leaders and discuss ways for them to improve. (C6) |
| The facilitator helped me to reflect and realise that I tended to take the easier option, avoid having those uncomfortable conversations because I would look for the perfect way to say it, think about the delivery too much and second guess myself, then the opportunity would be lost…we’ve had conversations about how to overcome that (P3) |
| Constantly reflecting, discussing where we are, where we want to be, reflecting on what's worked, talking about improvement areas, that’s on the facilitator, challenging us as leaders. (P7) |
| I'd get the leadership group in, win, lose, or draw, after the game, have a bit of a reflection straight up when it happened, just with the leaders. It might only be five minutes, but we just talk about the immediate response to the game, their leadership, because that's important because it's just happened. And you get a perspective on it that's a bit raw, and then off you go. A little honest reflection, and that can be a bit fraught, raw, then you can take a look at it. I've found that that's quite useful. It's easy to switch off after the game and not think about it at all, so I thought if we were clear about a couple of points that we were good at and a couple of points that may be improved, and they're embedded in our leader’s heads immediately after the game, then we revisit it in more depth on the Monday. (C4) |
| We debrief the task; asking **them** [athlete leaders] what was good, what was bad…how did you handle that, how did you think it went? (C8). |
| He [facilitator] challenges us a lot, which I think is terrific. And he does it in his own unique way, he’s really honest and upfront, which is exactly what we need ,he’ll hit us straight between the eyes and challenges us to really reflect on our leadership (P8) |
| We always found it helpful after those things [leadership development visits and activities] talking with the leadership guy about them and seeing what ways that we could incorporate whatever we learned moving forward. (P12) |
